# Development of UV spectrophotometry methods for concurrent quantification of amlodipine and celecoxib by manipulation of ratio spectra in pure and pharmaceutical formulation

**DOI:** 10.1371/journal.pone.0222526

**Published:** 2019-09-16

**Authors:** Mahesh Attimarad, Venugopla Katarigatta Narayanswamy, Bandar Essa Aldhubaib, Nagaraja SreeHarsha, Anroop Balachandran Nair

**Affiliations:** 1 Department of Pharmaceutical Sciences, College of Clinical Pharmacy, King Faisal University, Al Ahsa, KSA; 2 Department of Biotechnology and Food Technology, Durban University of Technology, Durban, South Africa; Institut d'Investigacions Biomediques de Barcelona, SPAIN

## Abstract

Recently, the United States Food and Drug Administration approved a new oral dosage preparation of amlodipine besylate (AML) and celecoxib (CEL) for the management of hypertension and osteoarthritis. However, no simultaneous estimation procedures for these two analytes have been described. Hence, two simple, accurate, and precise ultraviolet spectroscopic procedures that manipulated the ratio spectra were established for concurrent quantification of AML and CEL using ethanol as a solvent. The first method involves determining the peak-to-trough amplitude difference of the ratio spectra of AML and CEL. The second method involves determining the peak amplitude of the ratio first derivative (Δλ 4 nm) spectra of AML and CEL at 334.2 nm and 254.2 nm, correspondingly. Both methods showed linearity in the range of 1–6 μg mL-1 for AML and 5–40 μg mL-1 for CEL with an excellent correlation coefficient (<0.999). The proposed procedures were validated by following the International Conference on Harmonization guidelines for accuracy, precision, selectivity, recovery, and stability studies. It is evident from the low %RSD and %RE that both analytical procedures were found to be accurate and precise, respectively. The percent recovery of AML and CEL from the formulation was found to be 99.79% and 99.34% using the ratio-difference method and 100.13% and 99.70% using the ratio first-derivative method, with a low percent relative standard deviation. Further, the proposed techniques permit concurrent quantification of AML and CEL in different concentration ratios without interference from each other; hence, these techniques can be adopted for regular quality-control studies.

## Introduction

**Fig 1:**
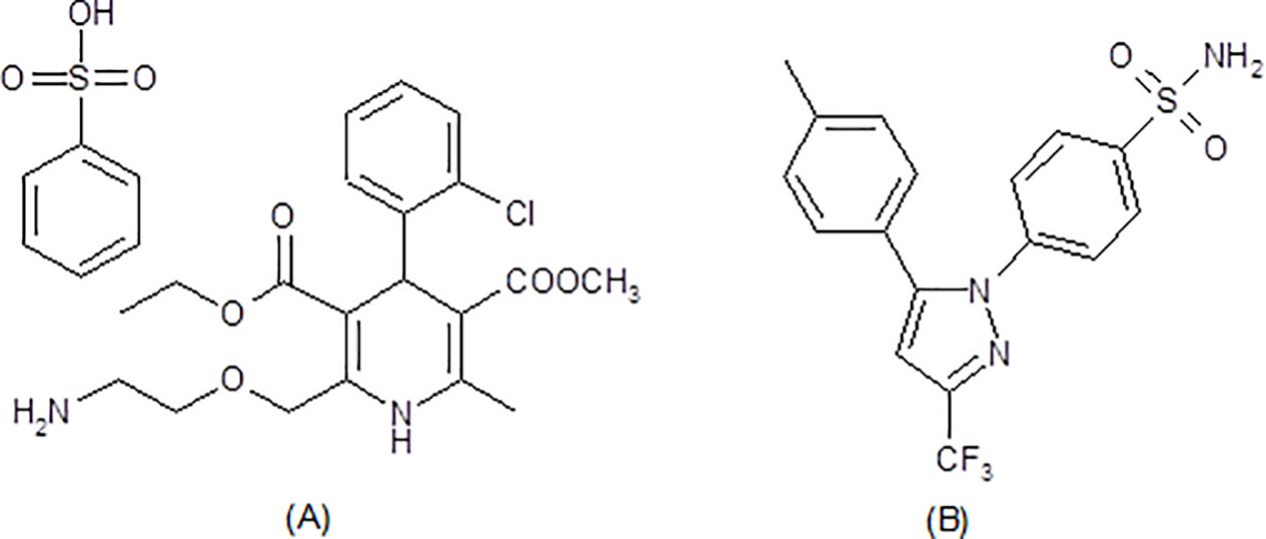
Chemical structures of amlodipine besylate (A) and celecoxib (B).

Middle-aged and older adults are suffering from many health problems, such as hypertension, diabetes, and osteoarthritis due to modern lifestyles and stress. Calcium-channel blockers are the most extensively prescribed medication in the management of hypertension due to its long duration of action. Amlodipine besylate (AML; Fig 1A), a dihydropyridine derivative, is a potent antihypertensive and antianginal drug that obstructs the migration of calcium ions in both vascular and cardiac muscles, thereby inhibiting the contraction of smooth and cardiac muscles. AML also reduces blood pressure via a peripheral vasodilation effect by reducing peripheral vascular resistance. [1,2]

Osteoarthritis is a degenerative disorder of the articular cartilage that leads to structural modifications of the bone. [3] Osteoarthritis may result from aging, obesity, and genetics. Those who are older and/or obese generally suffer from hypertension. Also, approximately 40% of those with hypertension are also diagnosed with osteoarthritis. [4–5] For the treatment of osteoarthritis-related pain, different nonsteroidal anti-inflammatory drugs (NSAIDs) were prescribed; however, for patients suffering from hypertension along with osteoarthritis, special attention should be paid when selecting NSAIDs, as most NSAIDs have an effect on blood pressure, and use of non-selective COX inhibitors can result in gastric ulceration. Conversely, the selective COX-2 inhibitor celecoxib (CEL; Fig 1B) has gastroprotective effects along with little effects on blood pressure. [6–7] As such, the United States Food and Drug Administration (FDA) has recently approved a fixed-dose oral formulation of AML and CEL for the management of hypertension and osteoarthritis. [8].

Different analytical techniques were described in the public domain for the quantification of AML from pharmaceutical preparations and plasma. AML alone has been estimated by spectrophotometric, [9, 10] fluorometric, [11] reverse-phase high-performance liquid chromatography (RP-HPLC), [12–14] and liquid chromatography–tandem mass spectrometry (LCMS/MS) methods [15]. AML, along with other antihypertensive drugs, were determined by spectrophotometric [16], high-performance thin-layer chromatography (HPTLC), [17] and electroanalytical methods [18–23]. Besides, few high-performance liquid chromatography (HPLC) procedures have been described for the concurrent estimation of AML with statins. [24,25]

Few spectroscopic procedures have been described in the estimation of CEL alone and various drug combinations. [26–28] Many chromatographic methods, along with stability-indicating procedures, were reported in the public domain for the concurrent estimation of CEL alone and in combination with other drug formulations and biological fluids. [29–33] Recently, the LCMS/MS method has been described for the quantification of CEL from human plasma. [34] In addition, the capillary zone electrophoresis (CZE) technique has been adopted for quantification of CEL in pharmaceutical formulations. [35,36]. However, no spectroscopic procedures have been reported for the concurrent quantification of AML and CEL in formulations. Therefore, it would be useful to establish simple, accurate, precise, and specific spectroscopic methods for the concurrent quantification of AML and CEL. The ultraviolet (UV) spectrophotometric method has been extensively used in analytical and clinical laboratories to analyze drugs in pharmaceutical formulations due to it’s simple, accurate, and reproducible results. However, many drugs have good UV absorption due to the presence of aromatic and heteroaromatic groups. Hence, multi-component formulations produce highly overlapped UV spectra, making it difficult to analyze the drugs directly in the presence of each other. Similarly, the ultraviolet (UV) spectrum of CEL completely overlaps that of AML, making it difficult to quantify both analytes by direct measurement. Hence, in the present work, we reported two ratio spectra manipulation spectroscopic procedures for the concurrent estimation of AML and CEL in a solid dosage form.

## Materials and methods

Instrumentation: A Shimadzu UV-Vis spectrophotometer (1600; Shimadzu Corporation, Kyoto, Japan) associated with the workstation was used for the development of the analytical methods. Then, 10 mm quartz cuvettes were used to measure solution absorbance. The slit width and scan speed were adjusted to 1 nm and medium, respectively. The manipulation of scanned spectra was performed using Shimadzu software (UV-Probe version 2.0; Shimadzu Corporation). Analytes and formulations were weighed using a digital balance.

Chemicals: Pure samples of AML and CEL were purchased from Sigma-Aldrich Co. (St. Louis, MO, USA). Analytical-grade ethanol was procured from Sigma Aldrich Co. Pure water was prepared using the Milli Q (Millipore, Billerica, MA, USA) water purification system, which was used throughout the experiments. AML (10 mg/tablet) and CEL (200 mg/capsule) were obtained from the pharmacy.

The tablets were prepared in a laboratory and included 200 mg CEL, 10 mg AML / 5 mg AML, 10 mg sodium starch glycolate type A, 12.5 mg talc, 10mg microcrystalline cellulose, 5 mg magnesium stearate and 8 mg lactose per tablet

### Preparation of standard solutions

To prepare the standard stock solutions of AML and CEL, 100 mg of AML and CEL were accurately weighed into two 100 mL measuring flasks, separately. Both analytes were dissolved using 60 mL of ethanol, and the final volumetric capacity reached 100 mL with the addition of ethanol. Standard solutions were kept in the refrigerator (4°C) until use. Standard solutions were used to prepare the calibration curve and for validation purposes, and laboratory-prepared solutions were arranged by transferring the required amount of standard solutions into 10 mL volumetric flasks.

### Preparing the sample solutions

AML and CEL combination tablets were not available in Saudi Arabia; hence, AML (10 mg) tablets and CEL (200 mg) capsules were used to prepare the samples. The weight of 20 AML tablets was determined; the average weight was computed, and the tablets were crushed into a smooth powder. Similarly, the powder of 20 CEL capsules was collected and the average weight was computed. AML powder corresponding to 50 mg of AML, as well as CEL capsule contents corresponding to 1,000 mg of CEL, were transferred and mixed thoroughly. Then, the powder mixture, corresponding to 10 mg of AML and 200 mg of CEL, was moved into a 100 mL measuring flask consisting of 60 mL of ethanol. The contents were dissolved with the help of ultrasonication (10 minutes). The solution was filtered using Whatman filter paper in a 100 mL measuring flask. The filter paper and remaining solution were rinsed with fresh ethanol; the final volume was adjusted up to the level with ethanol. The required amount of sample fraction was transferred into a 10 ml measuring flask and the ethanol was added to obtain the analyte quantity, which fell in the calibration range.

### Preparing the sample solutions using laboratory prepared tablets

Laboratory-prepared tablets were powdered, and powder equivalent to 10 mg of AML and 200 mg of CEL, as well as 5 mg of AML and 200 mg of CEL, was transferred separately into two conical flasks. Analytes were extracted by adding ethanol (30 ml x3). Each time solutions were sonicated for 10 min, filtered into 100 ml measuring flasks and the residue was washed with fresh ethanol. The final volume of both solutions were adjusted to 100 mL with ethanol. Further, solutions were diluted with ethanol to obtain a concentration in the range of the calibration curve.

### Procedure to prepare the calibration curve using ratio and first-derivative ratio spectra

The six working standard solutions comprising of 1–6 μg mL^-1^ (1, 2, 3, 4, 5, and 6 μg mL^-1^) of AML and 5–40 μg mL^-1^ (5, 10, 20, 25, 30, and 40 μg mL^-1^) of CEL were prepared by diluting the standard solutions with ethanol. AML and CEL solutions with a concentration 2 μg mL^-1^ and 10 μg mL^-1^, respectively, were arranged independently. UV absorption spectra were recorded for all solutions in the UV range of 200–400 nm using ethanol as a blank; the spectra were stored on a computer. Then, the ratio spectra were constructed by dividing the stored spectra by the CEL spectra of the 10 μg mL^-1^ solution; the ratio spectra were stored to perform the ratio-difference method. Further, the ratio spectra were converted into first-derivative spectra using 4 nm as Δλ. Similarly, the ratio spectra for CEL were constructed by dividing the combined spectra of the AML and CEL solutions by the AML spectra of the 2 μg mL^-1^ solution. Further, these spectra were converted into first-derivative spectra using 4 nm as Δλ. For the ratio-difference method, the peak amplitude difference was calculated by subtracting the amplitude of the trough at 345.6 nm from the amplitude of the peak at 336.4 nm for AML, and 236.5 nm from 266.8 nm for CEL. Then, the calibration curves were constructed by plotting a graph between the difference in peak amplitude with the corresponding concentrations of AML and CEL. Alternatively, regression equations were developed from the calibration curve.

For the first-ratio-derivative method, peak heights were determined at 334.2 nm for AML and 254.2 nm for CEL. Then, the calibration curves were created by drawing a graph between the peak amplitude and its relevant concentration. Alternatively, regression equations were developed from the calibration curve.

### Procedure to create laboratory mixed solutions

Nine mixed solutions were prepared in the laboratory by transferring the aliquot of AML and CEL stock solutions to obtain concentrations of 1:10, 2:10, 3:10, 1:20, 2:20, 3:20, 1:30, 2:30, and 3:30 μg mL^-1^, respectively. Normal UV spectra were recorded for all solutions in the UV range of 200–400 nm; these spectra were converted into ratio spectra and the first-derivative of ratio spectra. Amplitudes were determined by adopting the above procedure; concentrations were determined by the regression equation.

## Results and discussion

**Fig 2:**
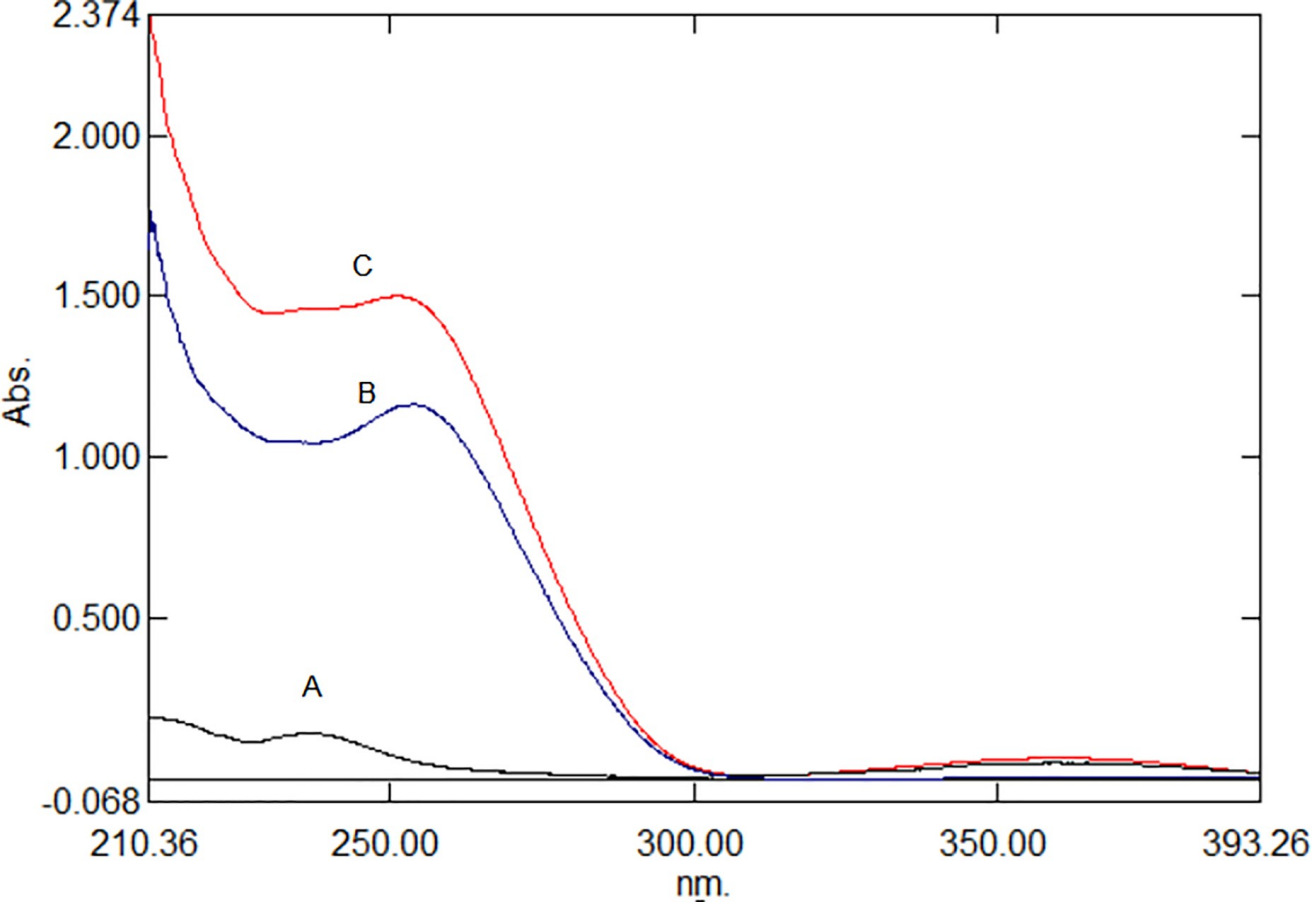
UV spectra of pure AML(A), CEL(B) and Formulation (C).

Normal absorption spectra for AML and CEL (Fig 2) showed complete overlap, which hindered the direct determination of these two analytes without prior separation. In such cases, the transformation of the UV absorption spectra into ratios and/or derivative ratio graphs can be adopted for the simultaneous determination of multicomponent formulations. [37, 38] The ratio spectra and their derived spectra permit the quantification of one component that is present alongside another component, as well as with excipients without their interference. [37–41]

### Theory of the ratio-absorbance-difference and ratio-derivatization techniques [37–41]

According to Beer’s law, the absorption of two components at a particular wavelength can be expressed by Eq 1:

$$AM=\varepsilon_{m}C_{m}+\varepsilon_{n}C_{n}$$ (1)

where AM is the absorbance of the combination of two components, m and, n; εm and εn are the molar absorptivity of components, m and n, at that particular wavelength; and Cm and Cn are concentrations of m and n, respectively. To cancel the absorbance of one of the components, Eq (1) has to be divided by an absorbance equation of one of the pure components (n) with a concentration n˚[*An˚ = εn˚ Cn˚*], which results in Eq (2):

$$\frac{AM}{An{}^{\circ}}=\frac{Am}{An{}^{\circ}}+\frac{Cn}{Cn{}^{\circ}}$$ (2)

The expression Cn/Cn˚ is constant (K); Eq (2) can be simplified into Eq (3):

$$P_{M}=P_{m}+K$$ (3)

where P_M_ is AM/An˚, the ratio absorption spectra of the mixture to component n˚; P_m_ is the Am/An˚ ratio absorbance spectra of component m to component n˚.

Constant K can be eliminated by determining the difference between the peak amplitude (ΔP) at two different wavelength points (λ1 and λ2), according to Eq (4):

$$\Delta P=P_{M1}-P_{M2}=\left(P_{1M}+K\right)-(P_{2M}+K)=P_{1M}-P_{2M}$$ (4)

where P_1m_ and P_2m_ represent the peak amplitudes of the ratio spectra at two wavelengths, λ1 and λ2, respectively.

Hence, the component n is canceled; the peak amplitude difference represents only component m. Component m can be determined from a mixture of components by constructing the calibration curve between the difference in peak amplitudes at two different wavelengths of the ratio spectra against the concentration of m. Similarly, following the same process, another component n can be determined in the presence of component m.

**Fig 3:**
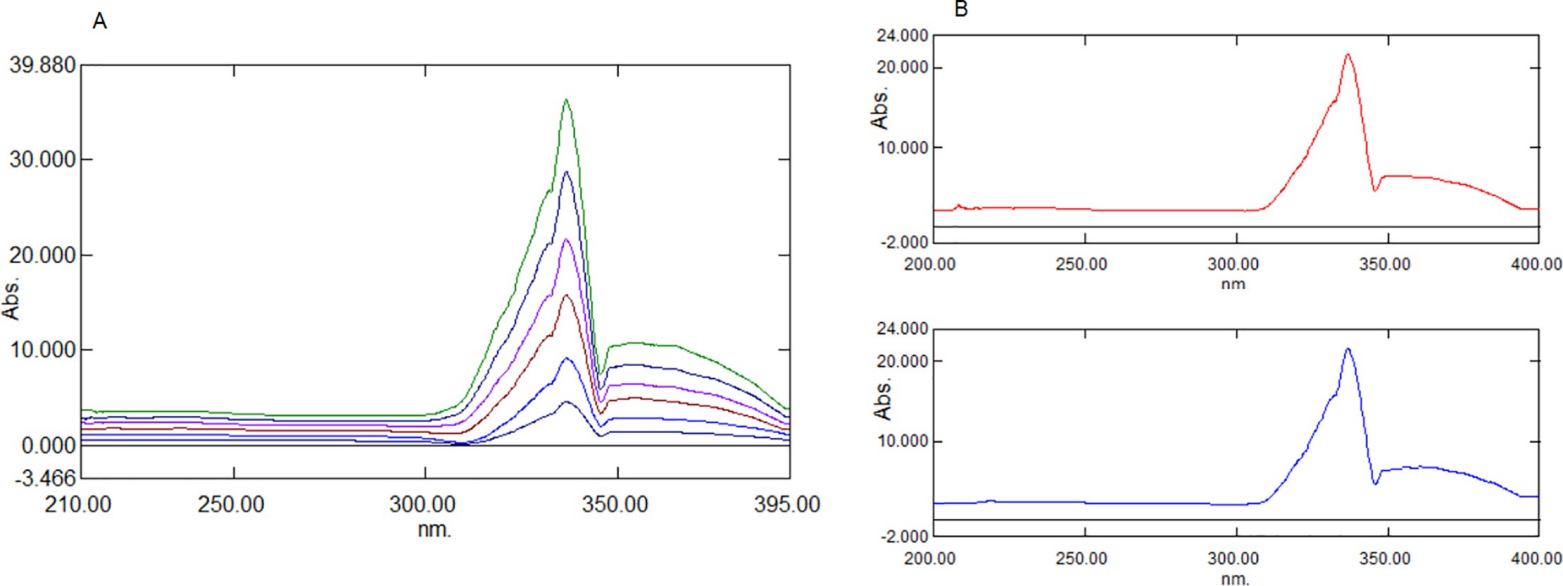
Ratio spectra of AML. (A) Ratio spectra of standard AML solutions (1–6 μg mL^-1^) using 10 μg mL^-1^ CEL as devisor. (B) Comparison of ratio spectra of pure AML and mixture of AML and CEL using 10 μg mL^-1^ CEL as devisor.

**Fig 4:**
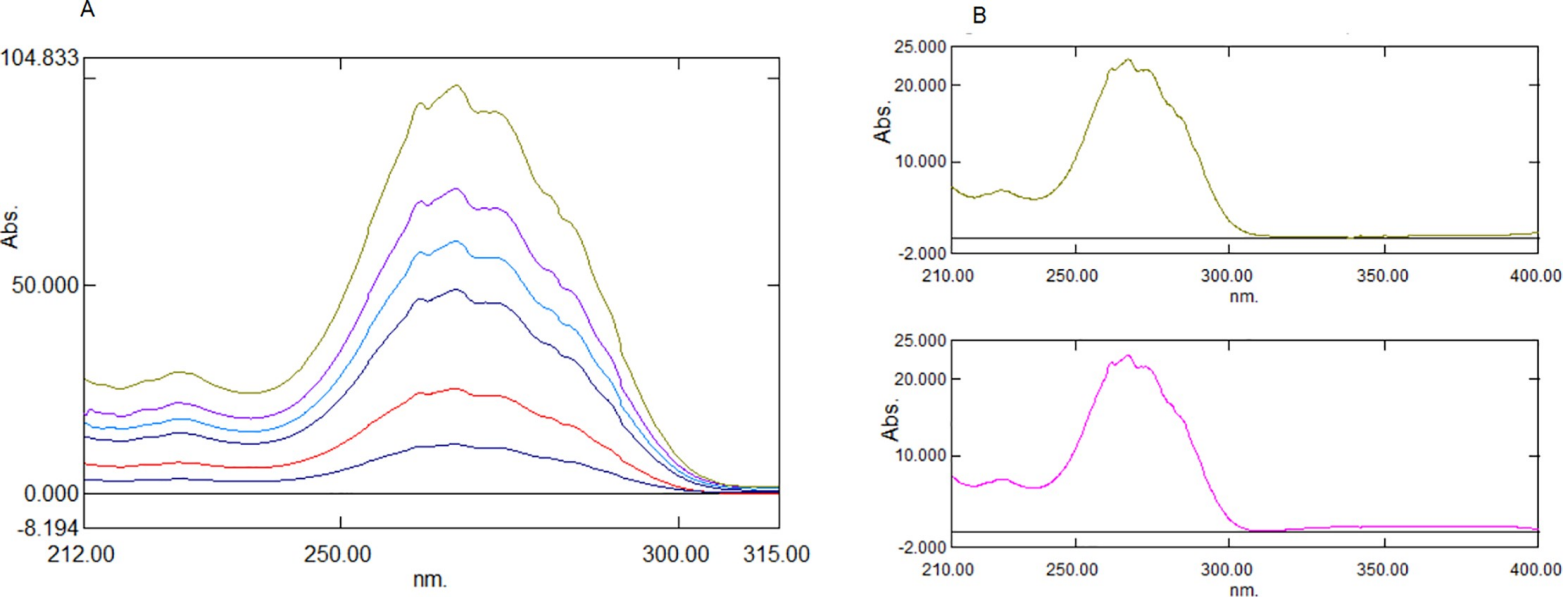
Ratio spectra of CEL. (A) Ratio spectra of standard CEL solutions (5–40 μg mL^-1^) using 2 μg mL^-1^ AML as devisor. (B) Comparison of ratio spectra of pure CEL and mixture of AML and CEL using 2 μg mL^-1^ AML as devisor.

In the present work, the ratio spectra of AML with different concentrations were obtained by scanning the mixture of AML and CEL in increased concentrations and dividing the normal spectra with the UV absorption spectra of CEL (10 μg mL^-1^; Fig 3A). The difference in the peak amplitude of the ratio spectra at a peak of 336.4 nm and trough of 345.6 nm was proportional to the concentration of AML. Further, this was supported by comparing the ratio spectra of the pure AML standard solution and the ratio spectra of the mixture of CEL, as well as with AML containing the same amount of AML. The constant difference between these two spectra was due to the Cn/Cn˚ in Eq (2). This unwanted parameter can be excluded by taking the variance between peak amplitudes measured at two wavelengths (336.4 nm and 345.6 nm). Generally, the two selected wavelengths should be the peak and trough of the ratio spectra to obtain maximum sensitivity. Fig 3B shows that the difference between the peak and trough amplitudes of the mixture and standard AML ratio spectra were the same; hence, the concentration of AML can be determined even in the presence of CEL. Similarly, CEL was determined by constructing the ratio spectra, which was achieved by dividing the normal spectra of the increased concentration of AML and CEL with the UV absorption spectra of the AML (2 μg mL^-1^) solution (Fig 4A). The peak height (266.8 nm) and trough (236.5 nm) of the ratio spectra were selected to measure the difference, which was proportional to the concentration of CEL. Fig 4B shows that the difference between the peak and trough amplitudes of the mixture and standard CEL ratio spectra were the same, irrespective of the AML concentration in the mixture.

### Ratio-first-derivative spectroscopic method

**Fig 5:**
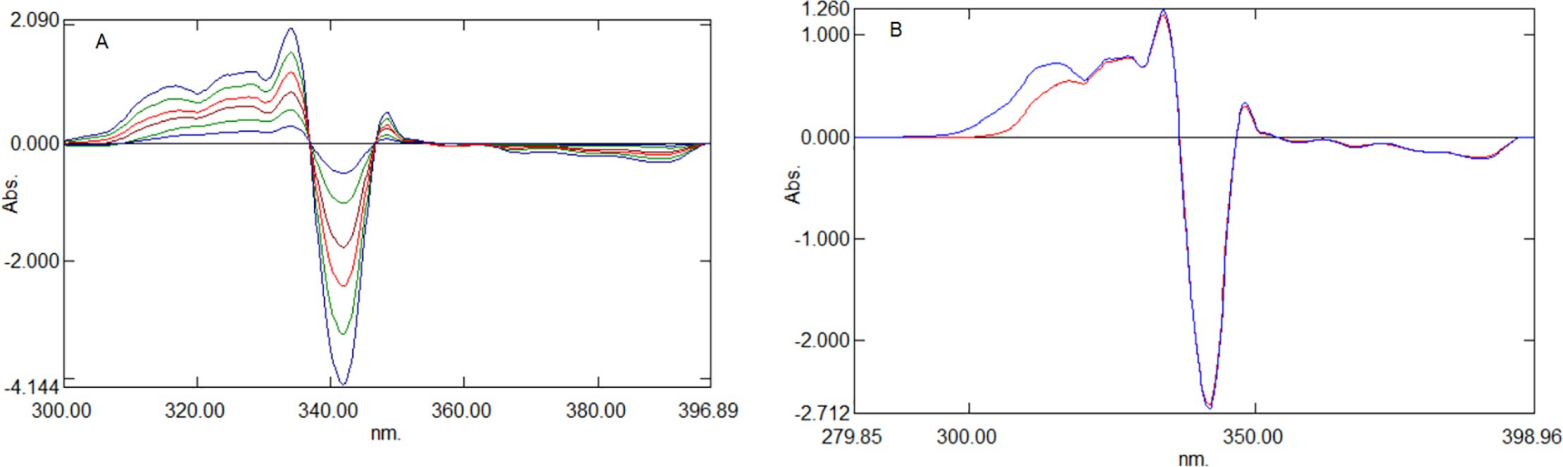
First derivative (Δλ 4nm) of ratio spectra of AML. (A) First derivative of standard AML solutions (1–6 μg mL^-1^) using 10 μg mL^-1^ CEL as devisor. (B) Comparison of first derivative of ratio spectra of pure AML and mixture of AML and CEL using 10 μg mL^-1^ CEL as devisor.

**Fig 6:**
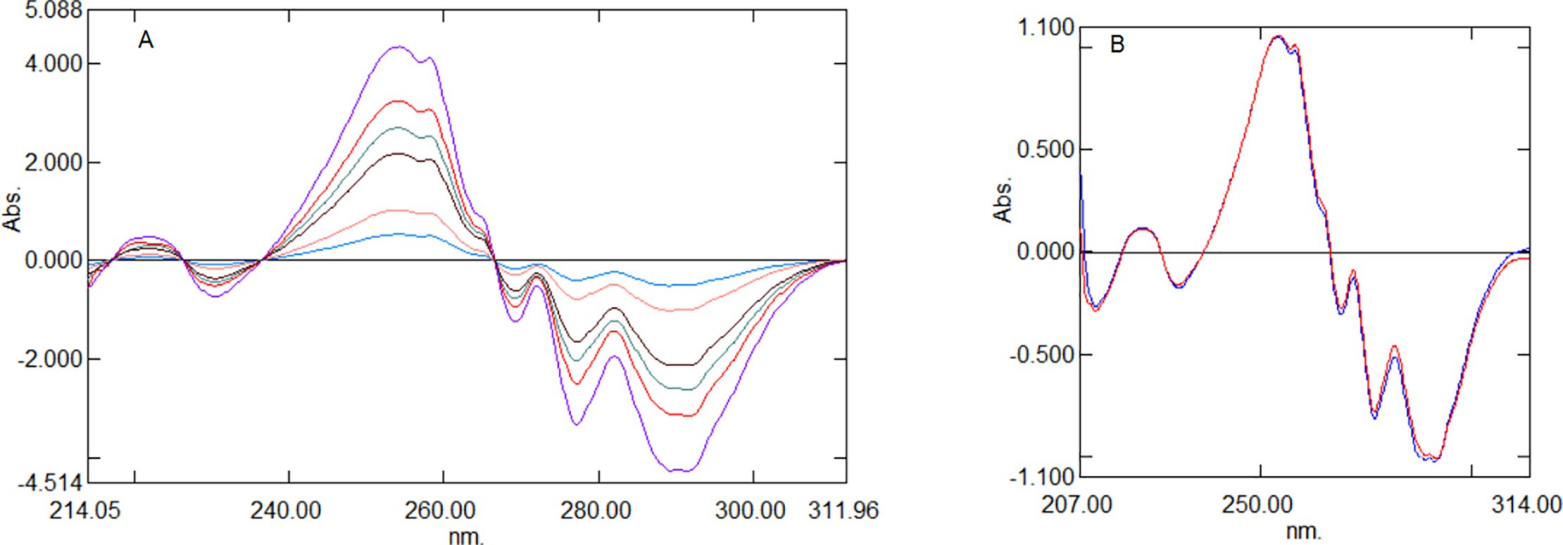
First derivative (Δλ 4nm) of ratio spectra of CEL. (A) First derivative of ratio spectra of standard solutions of CEL (5–40 μg mL^-1^) using 2 μg mL^-1^ AML as devisor. (B) Comparison of First derivative (Δλ 4nm) of ratio spectra of pure CEL and mixture of AML and CEL using 2 μg mL^-1^ AML as devisor.

As an alternative to the aforementioned method of measuring the difference between the peaks and troughs of the ratio spectra, derivatization of the ratio spectra can be adopted to resolve the interference spectra of binary mixtures. The derivatization of ratio spectra eliminates the constant interference value (Cn/Cn˚) in Eq (2) and provides many maxima and minima. This provides an opportunity to quantify one analyte in the presence of another analyte, as well as excipients, which generally cause interference during analysis. [38–41] In the second method, the ratio spectra of different concentrations were converted into first-derivative spectra using 4 nm as a derivatization wavelength (Δλ). Different wavelengths (2 nm, 4 nm, 8 nm, and 10 nm) were tested; however, 4 nm showed the most accurate results. The first-derivative ratio spectra of AML (Fig 5A) showed 2 minima at –342.2 nm and –390.2 nm, and four maxima at 316.6 nm, 327.9 nm, 334.2 nm, and 348.8 nm. Further, the peak amplitude at 316.54 nm, 327.9 nm, 348.70 nm, and –390.15 nm was lower, whereas the peak amplitude was satisfactory at 334.2 nm and –342.2 nm. Overall, 334.2 nm provided good linearity. Hence, a wavelength of 334.2 nm was selected for further processing. The first derivative of the ratio spectra of CEL (Fig 6A) showed two maxima at 222.0 nm and 254.2 nm, and four minima at –230.6 nm, –260.2, –277.2, and –291.4 nm. The peak amplitude at 222.0 nm, –230.6 nm, –260.2 nm, and –277.2 nm was lower, whereas good peak amplitude, linearity, and recovery were observed at 254.2 nm and –291.4 nm.

Also, a comparison of the first derivative of the ratio spectra for the combined AML and CEL, as well as the pure standard AML, showed the same amplitude at 334.2 nm, (Fig 5B) and whereas CEL showed the same amplitude at 254.2 nm (Fig 6B). Hence, wavelengths 334.2 nm and 254.2 nm were used to further develop the method.

### Method validation

Validation of the proposed manipulation of the UV-ratio spectroscopic methods was carried out in terms of linearity, the limit of detection, the limit of quantification, accuracy, precision, recovery studies, selectivity, and stability studies following International Conference on Harmonisation (ICH) guidelines.

Linearity: Linearity was established by converting the recorded UV absorption spectra into the ratio and first derivative of ratio spectra of both analytes. In both methods, AML and CEL showed good linearity with a concentration ranging from 1–6 μg mL^-1^ and 5–40 μg mL^-1^, correspondingly (S1 Fig). The linearity equations constructed from the standardization curve were tabulated in Table 1. In all cases, the correlation coefficient was found to be more than 0.999.

**Table 1:** Regression equations and validation parameters results for AML and CEL.

Parameters	Ratio difference method		Ratio first Derivative method	
Drugs	AML	CEL	AML	CEL
Wave length [nm]	336.4 345.6	266.8 236.5	334.2	254.2
Linearity Range [μg mL^-1^]	1–6	5–40	1–6	5–40
LOD [μg mL^-1^ ]	0.21	0.35	0.28	0.46
LOQ [μg mL^-1^]	0.65	0.97	0.83	1.27
Slop [m]	4.9135	1.8534	0.3154	0.1096
Intercept [c]	-2.2699	-0.4051	-0.0551	-0.0453
Correlation Coefficient [r^2^]	0.9994	0.9999	0.9991	0.9998

#### Limit of detection (LOD) and limit of quantification (LOQ)

As per the ICH guidelines, the LOD was calculated by 3.3 x/y and LOQ by 10 x/y. In these equations, x represents the standard deviation of the intercept and y represents the slope of the standard curve. The calculated LOD and LOQ are shown in Table 1. The small LOD and LOQ values describe the sensitivity of the projected methods.

#### Accuracy and precision

Intraday accuracy and precision were evaluated by investigating the laboratory-mixed analytes at three diverse concentration points covering the entire calibration range in triplicate on the same day. The same solutions were analyzed for three successive days to determine intraday accuracy and precision (S2 Fig). Precision was expressed as %RSD (Table 2) and it was <2%, demonstrating a high degree of precision for the procedures. Accuracy was expressed in terms of % relative error (Table 2). The results showed a low % relative error, indicating the excellent accuracy of the established procedures.

**Table 2:** Precision and accuracy data.

		Inter-day			Intra-day		
	Amount of Drug [μg mL^-1^]	Amount found Mean [n = 3] ± SD	%RSD	%RE	Amount found Mean [n = 9] ± SD	%RSD	%RE
Ratio difference method							
**AML**	1.00	0.99±0.01	1.01	-1.01	0.98±0.01	1.02	-2.04
	1.50	1.48±0.02	1.35	-1.35	1.51±0.03	1.99	0.66
	2.00	1.97±0.02	1.02	-1.52	1.98±0.02	1.01	-1.01
**CEL**	20.00	19.89±0.19	0.96	-0.55	20.11±0.15	0.75	0.55
	30.00	30.16±0.38	1.26	0.53	29.56±0.31	1.05	-1.49
	40.00	40.67±0.69	1.70	1.65	39.68±0.48	1.21	-0.81
Ratio first Derivative method							
**AML**	1.00	0.99±0.01	1.01	-1.01	1.01±0.02	1.98	0.99
	1.50	1.52±0.03	1.97	1.32	1.48±0.01	0.68	-1.35
	2.00	1.99±0.02	1.01	-0.50	2.01±0.04	1.99	0.50
**CEL**	20.00	19.91±0.24	1.21	-0.45	19.92±0.25	1.26	-0.40
	30.00	29.67±0.4	1.35	-1.11	29.81±0.33	1.11	-0.64
	40.00	39.56±0.63	1.59	-1.11	39.41±0.74	1.88	-1.50

SD: Standard deviation. %RSD: Percent Relative Standard Deviation. %RE: Percent Relative Error

#### Stability studies

The prepared stock solutions were stored in a refrigerator at 4°C. The stored solutions were analyzed daily; no difference was observed in the assay results, as determined by spectroscopic methods, which were compared to the day 1 results. Further, CEL was stable for 7 days, whereas AML began degrading after 5 days. This could be due to the instability of AML in the presence of light. [42–43].Hence, it is recommended that the solutions be covered with aluminum foil.

### AML and CEL determination from laboratory-prepared samples

Standard solutions contacting different concentrations of both analytes were mixed and analyzed (S3 Fig). The recovered drug concentrations (Table 3) of AML and CEL indicated the analytical power of the suggested spectroscopic procedures for the simultaneous determination of both analytes present in different ratios. Hence, these methods can be applied to analyze formulations comprising different ratios of AML and CEL (5:200, and 10:200).

**Table 3:** Summary of determination AML and CEL from laboratory mixed solutions by the proposed methods.

Concentration taken		Percentage recovery ± SD*			
AML	CEL	Ratio Difference method		Ratio First derivative method	
		AML	CEL	AML	CEL
1	20	99.86±0.96	100.67±1.41	98.56±0.98	100.59±1.09
1	30	101.56±0.85	100.89±0.96	101.55±1.18	98.96±0.79
1	40	99.54±1.67	99.01±0.82	100.7±1.03	99.16±1.02
2	20	98.75±0.99	98.79±0.77	99.72±0.97	100.56±0.93
2	30	98.39±0.81	99.12±1.06	100.43±0.89	99.14±0.83
2	40	99.04±0.69	101.81±1.29	99.44±0.68	98.51±1.23
3	20	98.08±0.76	100.49±0.93	98.57±0.92	101.29±1.46
3	30	99.46±0.58	98.39±0.86	99.19±1.24	100.91±0.85
3	40	101.57±1.28	99.46±0.97	100.25±1.63	98.09±1.09
Mean	99.58	99.85		99.82	99.69
Mean %RSD	1.26	1.25		1.00	1.15

*SD: Standard deviation, Average of three determination, %RSD: Percent relative Standard deviation

#### Selectivity

The selectivity of the established procedures was confirmed by the results of the laboratory-mixed AML and CEL solutions at different concentrations. Acceptable results were achieved in the different ratios, as tabulated in Table 3. Further, the effect of tablet excipients was investigated by analyzing the tablet excipients without the analytes. The excipients did not show any absorbance in the applied wavelength for both methods.

### Determining AML and CEL in formulations

Established procedures were successfully employed for the concurrent quantification of AML and CEL from the pharmaceutical preparation. The outcomes of the investigation are presented in Table 4. The present assays for AML were 99.38% and 98.96%, whereas, for CEL, they were 101.4% and 99.17%. Across mean recovery studies conducted using the standard addition method (S4 Fig), the ratio difference was found to be 100.13% and 99.79%, while the ratio-first-derivative methods yielded values of 99.70% and 99.34% for AML and CEL, respectively. The low %RSD showed the degree of precision and reproducibility of the methods. The analysis results obtained from the laboratory—prepared tablet also showed that the methods used were highly accurate (Table 4, S5 Fig) The analysis and recovery results indicated that the accuracy of the methods, as well as the absence of formulation excipients interfere with the quantification of both analytes.

**Table 4:** Determination of AML and CEL from formulations and recovery studies by standard addition method.

	Ratio difference method		Ratio first Derivative method	
	Amount in [μg mL^-1^]	% Recovery	Amount in [μg mL^-1^]	% Recovery
Formulation [AML]	1	99.38	1	98.96
Formulation [CEL]	20	101.04	20	99.17
Tablet 10:200				
AML	1	98.57	1	101.43
CEL	20	99.08	20	99.49
Tablet 5:200				
AML	1	100.86	1	101.27
CEL	40	98.53	40	98.19
Recovery of added AML	0.5	99.67	0.5	99.39
	1	101.83	1	100.82
	1.5	98.89	1.5	98.90
	Across Mean	100.13		99.70
	%RSD	1.53		1.00
Recovery of added CEL	5	99.05	5	98.70
	10	100.56	10	99.08
	15	99.75	15	100.25
	Across Mean	99.79		99.34
	%RSD	0.76		0.81

## Conclusions

Overall, it was found that the two proposed UV spectrophotometric procedures–ratio difference and the first derivative of the ratio spectra–could simultaneously estimate AML and CEL in pure and pharmaceutical preparations with excellent accuracy and precision. However, the absorbance difference between ratio spectra has its advantages over the ratio-derivative method in terms of simplicity, due to the involvement of only two steps (the division and measurement of absorbance differences) when compared to the three steps required for the ratio-derivative method (division, derivatization, and absorbance measurement). Further, both methods are simple, fast, sensitive, and specific; they work without applying complicated equations or separation procedures. Hence, these techniques can be utilized to perform regular quality control studies of solid dosage forms that have different ratios of AML and CEL.

## Supporting information

S1 Fig Calibration curve for ratio difference method AML (A), CEL (B) and ratio first derivative method AML (C), CEL (D).
(DOCX)

S2 Fig Ratio and first derivative ratio spectra of CEL and AML for accuracy and precision studies. (A) Ratio spectra of CEL 20,30,40 μg ML^-1^ using 2 μg mL^-1^ solution spectra of AML. (B) First derivative of Ratio spectra of CEL 20,30,40 μg ML^-1^ using 2 μg mL^-1^ solution spectra of AML.(C) Ratio spectra of AML 1, 1.5, 2 μg mL^-1^ using 10 μg mL^-1^ solution spectra of CEL.(D) First Derivative of ratio spectra of AML 1, 1.5, 2 μg mL^-1^ for precision and accuracystudies using 10 μg mL^-1^ solution spectra of CEL for accuracy and precision studies.
(DOCX)

S3 Fig UV absorption spectra of laboratory prepared solutions of AML and CEL in different ratios (AML: CEL, 1:20, 1.5:20, 2:20; 1:30, 1.5:30, 2:30; and 1:40, 1.5:40, 2:40 μg ml-1 respectively). (DOCX)

S4 Fig Ratio and first derivative ratio spectra of CEL and AML for recovery studies. (A) Ratio spectra of CEL 20, 25, 30 35 μg ml^-1^ using AML 2 μg ml^-1^. (B) First derivative (Δλ 4 nm) of ratio spectra of CEL 20, 25, 30 35 μg ml^-1^ using AML 2 μg ml^-1^. (C) Ratio spectra of AML 1, 1.5, 2, 2.5 μg ml^-1^ using CEL 10 μg ml^-1^. (D) First derivative (Δλ 4 nm) of ratio spectra of AML 1, 1.5, 2, 2.5 μg ml^-1^ using CEL 10 μg ml^-1^ for recovery studies.
(DOCX)

S5 Fig Manipulated UV spectra of tablet solutions. Ratio spectra (A) and First derivative (Δλ 4 nm) of ratio spectra (B) of AML: CEL 1:20 and 1:40 μg ml^-1^ tablet solution using AML 2 μg ml^-1^ as divisor, Ratio spectra (C) and First derivative (Δλ 4 nm) of ratio spectra (D) of AML: CEL 1:20 AND 1:40 μg ml^-1^ tablet solution using CEL 10 μg ml^-1^ as divisor.
(DOCX)
