# Correction: Development of UV spectrophotometry methods for concurrent quantification of amlodipine and celecoxib by manipulation of ratio spectra in pure and pharmaceutical formulation

**DOI:** 10.1371/journal.pone.0227412

**Published:** 2019-12-30

**Authors:** Mahesh Attimarad, Katharigatta Narayanaswamy Venugopala, Bandar Essa Aldhubaib, Nagaraja SreeHarsha, Anroop Balachandran Nair

The second author's name is spelled incorrectly. The correct name is: Katharigatta Narayanaswamy Venugopala. The correct citation is: Attimarad M, Venugopala KN, Aldhubaib BE, SreeHarsha N, Nair AB (2019) Development of UV spectrophotometry methods for concurrent quantification of amlodipine and celecoxib by manipulation of ratio spectra in pure and pharmaceutical formulation. PLoS ONE 14(9): e0222526. https://doi.org/10.1371/journal.pone.0222526
