# Supplementary material for: Development of UV spectrophotometry methods for concurrent quantification of amlodipine and celecoxib by manipulation of ratio spectra in pure and pharmaceutical formulation
Source: PLoS One. 2019 Sep 16;14(9):e0222526. doi: 10.1371/journal.pone.0222526 (PMC6746368; doi:10.1371/journal.pone.0222526)
Supplement: S1 Fig — Calibration curve for ratio difference method AML (A), CEL (B) and ratio first derivative method AML (C), CEL (D). (DOCX) [file pone.0222526.s001.docx]

**
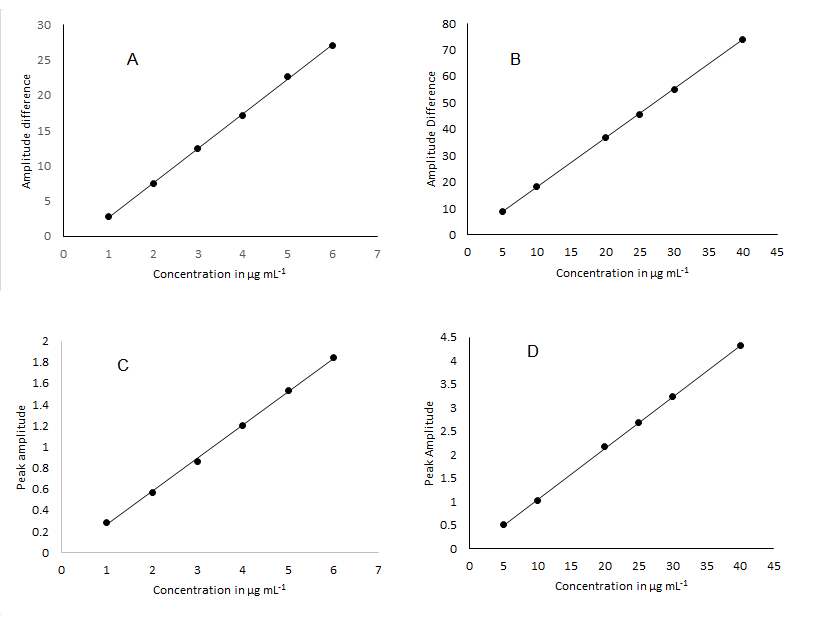
**

**S1 Fig. Calibration curve for ratio difference method AML (A), CEL (B) and ratio first derivative method AML (C), CEL (D)**
