# Supplementary material for: Development of UV spectrophotometry methods for concurrent quantification of amlodipine and celecoxib by manipulation of ratio spectra in pure and pharmaceutical formulation
Source: PLoS One. 2019 Sep 16;14(9):e0222526. doi: 10.1371/journal.pone.0222526 (PMC6746368; doi:10.1371/journal.pone.0222526)
Supplement: S2 Fig — (A) Ratio spectra of CEL 20,30,40 μg ML-1 using 2 μg mL-1 solution spectra of AML. (B) First derivative of Ratio spectra of CEL 20,30,40 μg ML-1 using 2 μg mL-1 solution spectra of AML.(C) Ratio spectra of AML 1, 1.5, 2 μg mL-1 using 10 μg mL-1 solution spectra of CEL.(D) First Derivative of ratio spectra of AML 1, 1.5, 2 μg mL-1 for precision and accuracystudies using 10 μg mL-1 solution spectra of CEL for accuracy and precision studies. (DOCX) [file pone.0222526.s002.docx]

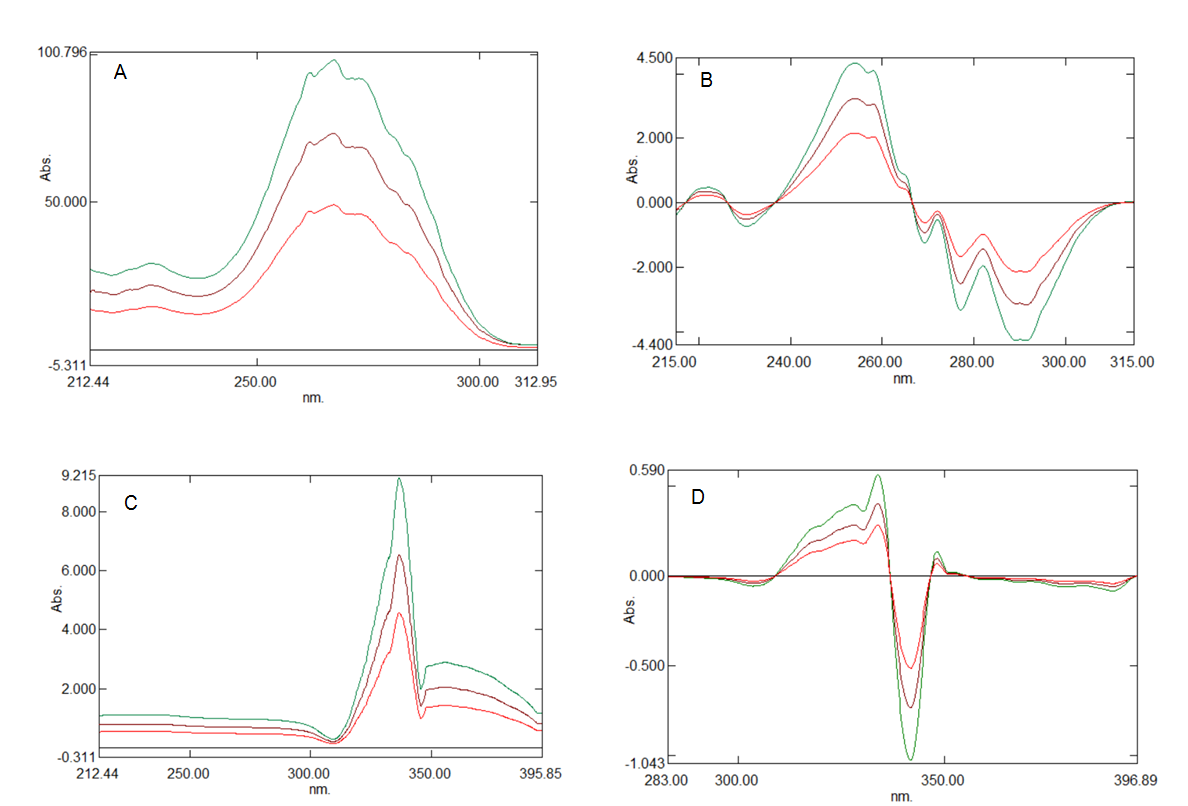


**S2 Fig. Ratio and first derivative ratio spectra of CEL and AML for accuracy and precision studies.**

(A) Ratio spectra of CEL 20,30,40 µg ML^-1^ using 2 µg mL^-1^ solution spectra of AML. (B) First derivative of Ratio spectra of CEL 20,30,40 µg ML^-1^ using 2 µg mL^-1^ solution spectra of AML.(C) Ratio spectra of AML 1, 1.5, 2 µg mL^-1^ using 10 µg mL^-1^ solution spectra of CEL.(D) First Derivative of ratio spectra of AML 1, 1.5, 2 µg mL^-1^ for precision and accuracystudies using 10 µg mL^-1^ solution spectra of CEL for accuracy and precision studies.
