# Supplementary material for: Development of UV spectrophotometry methods for concurrent quantification of amlodipine and celecoxib by manipulation of ratio spectra in pure and pharmaceutical formulation
Source: PLoS One. 2019 Sep 16;14(9):e0222526. doi: 10.1371/journal.pone.0222526 (PMC6746368; doi:10.1371/journal.pone.0222526)
Supplement: S3 Fig — (DOCX) [file pone.0222526.s003.docx]

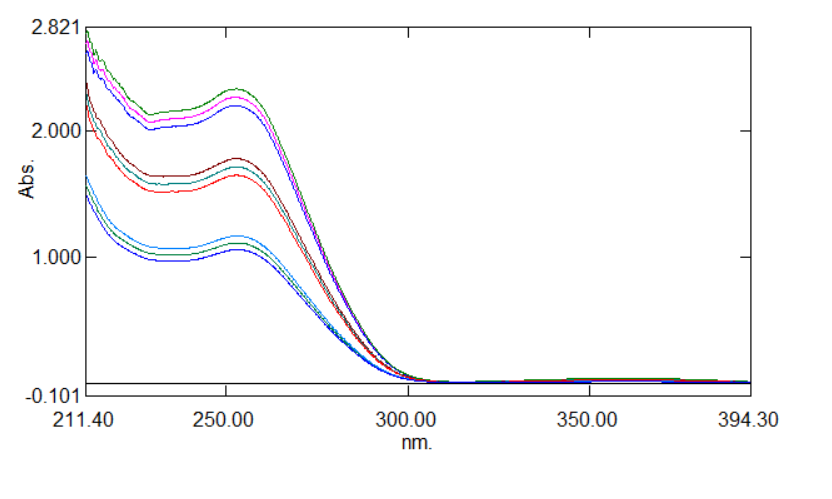


**S3 Fig. UV absorption spectra of laboratory prepared solutions of AML and CEL in different ratios (AML : CEL, 1:20, 1.5:20, 2:20; 1:30, 1.5:30, 2:30; and 1:40, 1.5:40, 2:40 µg ml^-1^ respectively)**
