# Supplementary material for: Development of UV spectrophotometry methods for concurrent quantification of amlodipine and celecoxib by manipulation of ratio spectra in pure and pharmaceutical formulation
Source: PLoS One. 2019 Sep 16;14(9):e0222526. doi: 10.1371/journal.pone.0222526 (PMC6746368; doi:10.1371/journal.pone.0222526)
Supplement: S4 Fig — (A) Ratio spectra of CEL 20, 25, 30 35 μg ml-1 using AML 2 μg ml-1. (B) First derivative (Δλ 4 nm) of ratio spectra of CEL 20, 25, 30 35 μg ml-1 using AML 2 μg ml-1. (C) Ratio spectra of AML 1, 1.5, 2, 2.5 μg ml-1 using CEL 10 μg ml-1. (D) First derivative (Δλ 4 nm) of ratio spectra of AML 1, 1.5, 2, 2.5 μg ml-1 using CEL 10 μg ml-1 for recovery studies. (DOCX) [file pone.0222526.s004.docx]

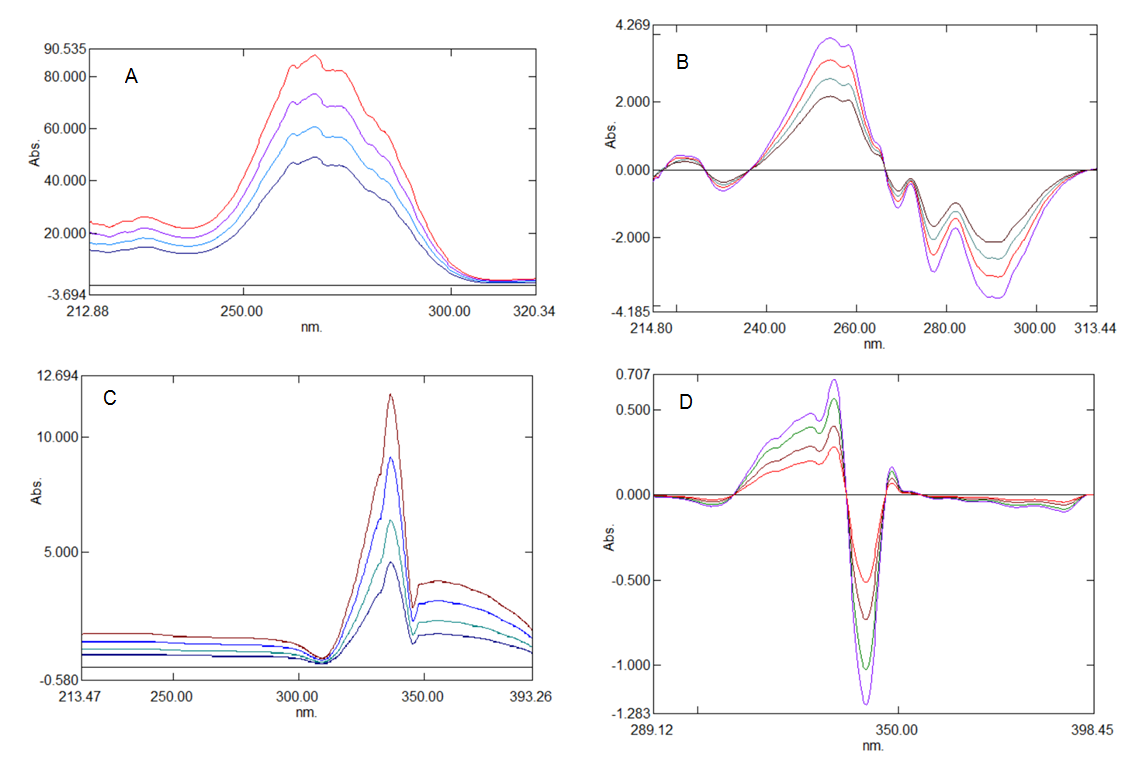


**S4 Fig. Ratio and first derivative ratio spectra of CEL and AML for recovery studies.**

(A) Ratio spectra of CEL 20, 25, 30 35 µg ml^-1^ using AML 2 µg ml^-1^. (B) First derivative (∆λ 4 nm) of ratio spectra of CEL 20, 25, 30 35 µg ml^-1^ using AML 2 µg ml^-1^. (C) Ratio spectra of AML 1, 1.5, 2, 2.5 µg ml^-1^ using CEL 10 µg ml^-1^. (D) First derivative (∆λ 4 nm) of ratio spectra of AML 1, 1.5, 2, 2.5 µg ml^-1^ using CEL 10 µg ml^-1^ for recovery studies.
