# Supplementary material for: Development of UV spectrophotometry methods for concurrent quantification of amlodipine and celecoxib by manipulation of ratio spectra in pure and pharmaceutical formulation
Source: PLoS One. 2019 Sep 16;14(9):e0222526. doi: 10.1371/journal.pone.0222526 (PMC6746368; doi:10.1371/journal.pone.0222526)
Supplement: S5 Fig — Ratio spectra (A) and First derivative (Δλ 4 nm) of ratio spectra (B) of AML: CEL 1:20 and 1:40 μg ml-1 tablet solution using AML 2 μg ml-1 as divisor, Ratio spectra (C) and First derivative (Δλ 4 nm) of ratio spectra (D) of AML: CEL 1:20 AND 1:40 μg ml-1 tablet solution using CEL 10 μg ml-1 as divisor. (DOCX) [file pone.0222526.s005.docx]

**
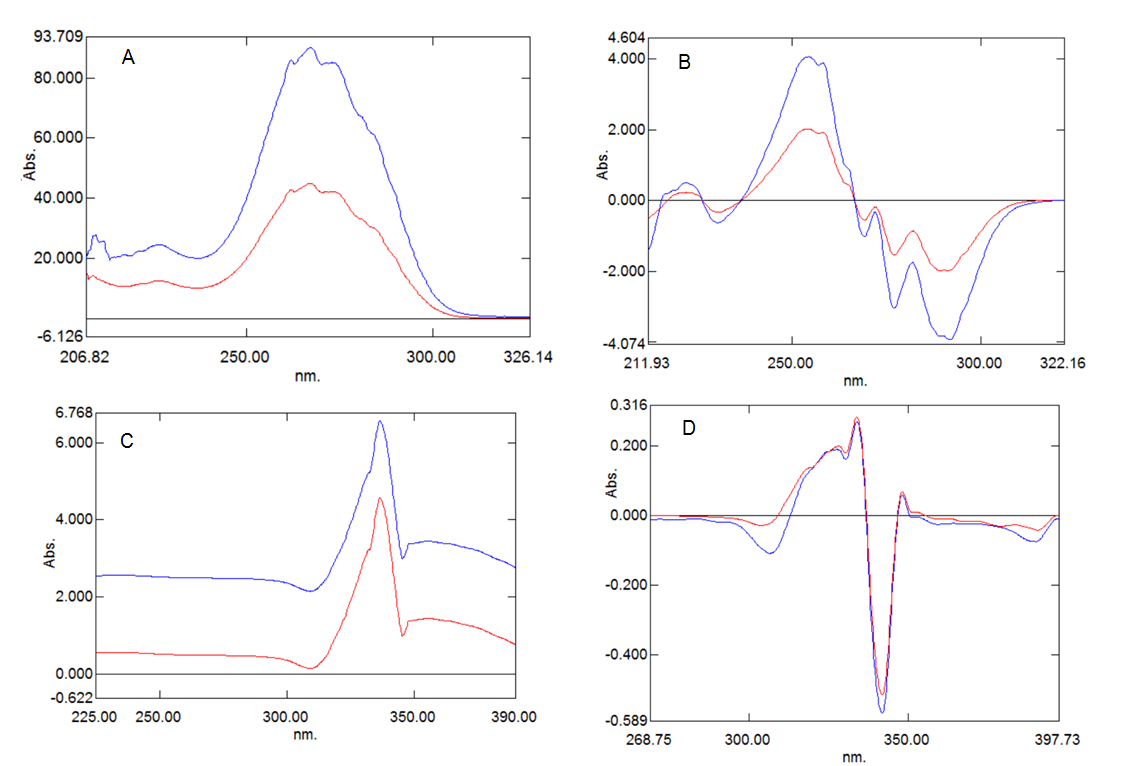
**

**S5 Fig. Manipulated UV spectra of tablet solutions**

Ratio spectra (A) and First derivative (∆λ 4 nm) of ratio spectra (B) of AML: CEL 1:20 and 1:40 µg ml^-1^ tablet solution using AML 2 µg ml^-1^ as divisor, Ratio spectra (C) and First derivative (∆λ 4 nm) of ratio spectra (D) of AML: CEL 1:20 AND 1:40 µg ml^-1^ tablet solution using CEL 10 µg ml^-1^ as divisor.
